# First person – Nikita Jhaveri

**DOI:** 10.1242/bio.062299

**Published:** 2025-10-23

## Abstract

First Person is a series of interviews with the first authors of a selection of papers published in Biology Open, helping researchers promote themselves alongside their papers. Nikita Jhaveri is first author on ‘ Coincident evolution and functional adaptation of the taxonomically-restricted genes *ivph-3* and *gon-14* in *Caenorhabditis* nematodes’, published in BiO. Nikita conducted the research described in this article while a PhD student in Bhagwati Gupta's lab at McMaster University, Hamilton, Canada. She is now a post-doctoral researcher in the lab of Erik Andersen at Johns Hopkins University, investigating how genetic variation shapes adaptation to different environments.


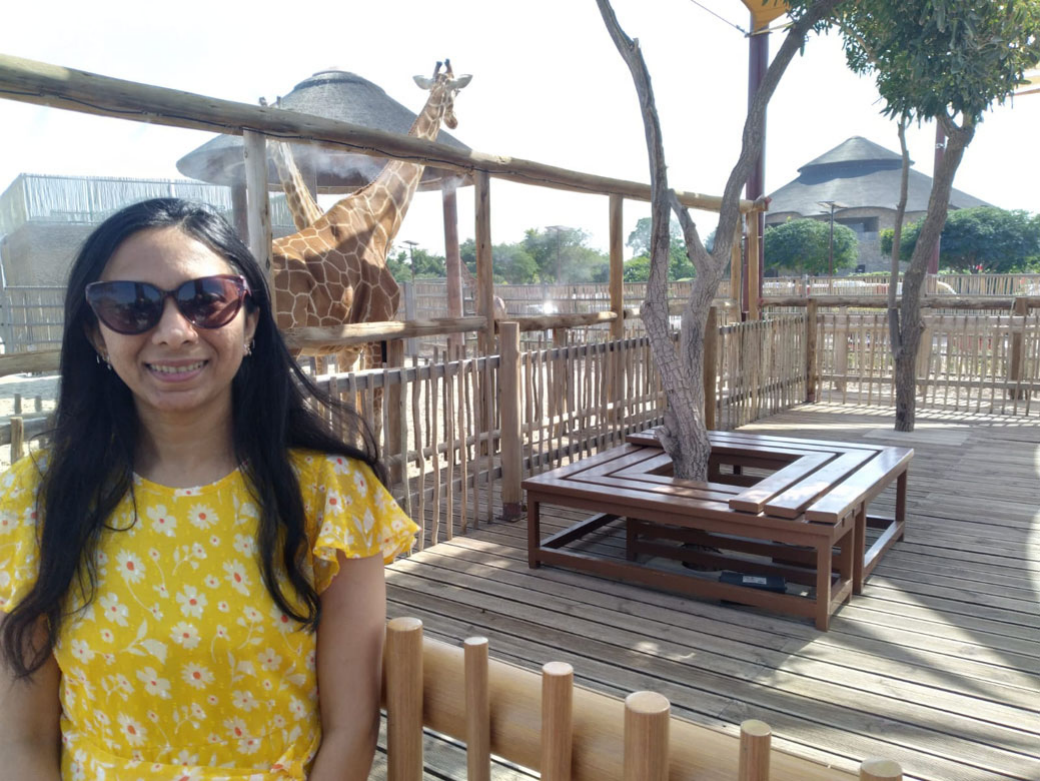


**Nikita Jhaveri**

**Describe your scientific journey and your current research focus**

My scientific journey began with studying negative regulators of cell proliferation, where I compared the function of these genes in closely related species and uncovered novel mechanisms of regulation. I then shifted my focus to understanding how organisms adapt to thermal stress, which led me to broader questions about local adaptation. Building on this, my current postdoctoral research focuses on investigating local adaptation in *Caenorhabditis* species, exploring how genetic and physiological variation contributes to survival and reproduction in different environments.

**Who or what inspired you to become a scientist?**

One of the first things that truly captivated me was learning how incredibly long DNA is and how it's packed so neatly into the tiny cells of our bodies. The idea that something so vast and complex could be organized on such a microscopic scale fascinated me and sparked my curiosity about biology. It made me realize that there's an entire world of intricate processes happening inside us that we are not aware of, yet they determine our lives. That sense of awe - recognizing the hidden complexity of life was one of the key inspirations for me to become a scientist. For me, science is about facts and uncovering the elegant systems that make life possible.

For me, science is about facts and uncovering the elegant systems that make life possible.

**How would you explain the main finding of your paper?**

Genes that are unique to certain round worms, like *ivph-3* and *gon-14*, can become essential for their survival and development. While these genes work similarly within a species, their effects can differ between species, showing that species-specific genes can adapt in different ways to ensure survival. This means that evolution doesn't just change existing genes – it can also create new genes important for how a species lives and adapts.

**What are the potential implications of this finding for your field of research?**

Our findings imply that taxonomically-restricted genes like *ivph-3* and *gon-14*, although poorly conserved across species, play essential and species-specific roles. Their functional divergence between two closely related species - *C. elegans* and *C. briggsae* indicate that lineage-specific genes may contribute to differences in traits. Moreover, because *ivph-3* broadly affects gene regulation, these genes may illustrate how new genes integrate into existing regulatory networks to influence key biological processes.

**Figure:**
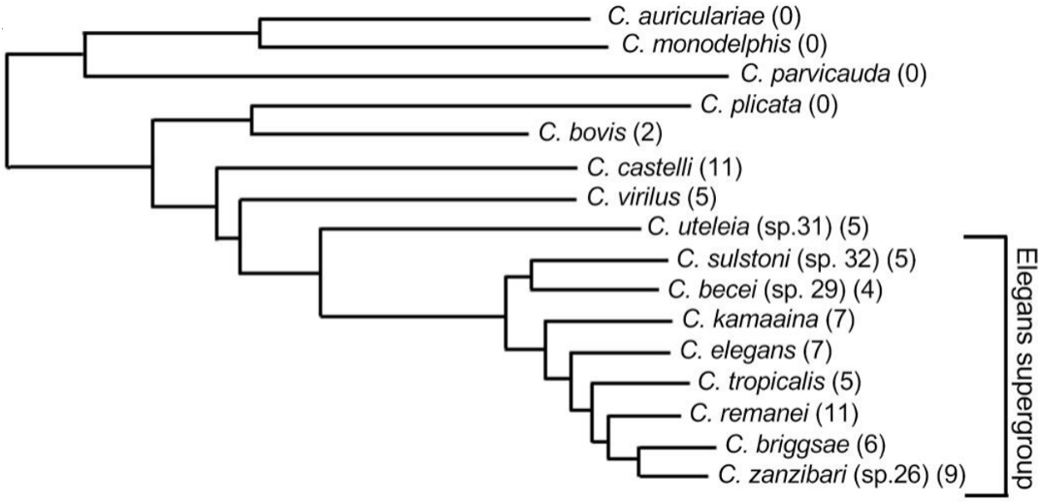
At the bench, uncovering how tiny worms reveal big secrets about adaptation.

**Which part of this research project was the most rewarding?**

The most rewarding part of this research project was collaborating with Dr Helen Chamberlin's lab, which gave me the opportunity to learn a new technique in a different research environment. I gained hands-on experience with CRISPR-Cas9–mediated gene editing, and I was thrilled when I successfully edited my first gene.

**What do you enjoy most about being an early-career researcher?**

What I enjoy most about being an early-career researcher is the balance of independence and mentorship. My PIs are incredibly supportive, allowing me to pursue my own ideas and explore new directions while providing guidance when needed. I also appreciate that I don't have to worry about securing grants or funding, which lets me focus fully on the science and on developing my skills. This combination of freedom, mentorship, and focus on research makes the experience both challenging and deeply rewarding.

**What piece of advice would you give to the next generation of researchers?**

When you begin your research, it's natural to feel excited and inspired by ideas. The excitement can drive you, but the true challenge lies in staying consistent and disciplined over the long term. Experiments will often fail, results may be unexpected, and setbacks can feel discouraging – but these are not signs of personal failure. Every unexpected result is an opportunity to learn something new and to refine your approach. Embrace the process, remain curious, and let persistence guide you, because resilience and consistency are just as important as creativity in shaping a successful scientific journey.

the true challenge lies in staying consistent and disciplined over the long term

**What's next for you?**

I am currently a postdoctoral researcher in Erik Andersen's lab at Johns Hopkins University, USA, and I am eager to transition into an industry role where I can leverage my expertise and skills to drive innovation and contribute to the advancement of the biotechnology sector.
